# Crystal structure of the catalytic D2 domain of the AAA+ ATPase p97 reveals a putative helical split‐washer‐type mechanism for substrate unfolding

**DOI:** 10.1002/1873-3468.13667

**Published:** 2019-11-22

**Authors:** Lasse Stach, Rhodri Marc Morgan, Linda Makhlouf, Alice Douangamath, Frank von Delft, Xiaodong Zhang, Paul S. Freemont

**Affiliations:** ^1^ Section of Structural and Synthetic Biology Department of Infectious Disease Imperial College London UK; ^2^ Centre for Structural Biology Department of Life Sciences Imperial College London UK; ^3^ Diamond Light Source Ltd Harwell Science and Innovation Campus Didcot UK; ^4^ Structural Genomics Consortium Nuffield Department of Medicine University of Oxford UK; ^5^ Department of Biochemistry University of Johannesburg Auckland Park South Africa

**Keywords:** AAA+ ATPase, D2 domain, IBMPFD, p97

## Abstract

Several pathologies have been associated with the AAA+ ATPase p97, an enzyme essential to protein homeostasis. Heterozygous polymorphisms in p97 have been shown to cause neurological disease, while elevated proteotoxic stress in tumours has made p97 an attractive cancer chemotherapy target. The cellular processes reliant on p97 are well described. High‐resolution structural models of its catalytic D2 domain, however, have proved elusive, as has the mechanism by which p97 converts the energy from ATP hydrolysis into mechanical force to unfold protein substrates. Here, we describe the high‐resolution structure of the p97 D2 ATPase domain. This crystal system constitutes a valuable tool for p97 inhibitor development and identifies a potentially druggable pocket in the D2 domain. In addition, its P6_1_ symmetry suggests a mechanism for substrate unfolding by p97.

**Database:**

The atomic coordinates and structure factors have been deposited in the PDB database under the accession numbers http://www.rcsb.org/pdb/search/structidSearch.do?structureId=6G2V, http://www.rcsb.org/pdb/search/structidSearch.do?structureId=6G2W, http://www.rcsb.org/pdb/search/structidSearch.do?structureId=6G2X, http://www.rcsb.org/pdb/search/structidSearch.do?structureId=6G2Y, http://www.rcsb.org/pdb/search/structidSearch.do?structureId=6G2Z and http://www.rcsb.org/pdb/search/structidSearch.do?structureId=6G30.

## Graphical abstract

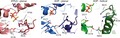

## Abbreviations

**FL**, full‐length

**IBMPFD**, inclusion body myopathy with early‐onset Paget disease and frontotemporal dementia

**ITC**, isothermal titration calorimetry

**VAT**, VCP‐like ATPase

The AAA+ ATPase p97 (valosin‐containing protein VCP) is a highly abundant enzyme with essential roles in multiple cellular processes 1. It was initially identified as an Mg+(2)‐ATPase with sixfold symmetry and an apparent molecular weight of 97 kDa, homologous to yeast cell division protein cdc48 2, 3. The enzyme was subsequently shown to play a role in various cellular processes, both nuclear and cytoplasmic, reviewed by Meyer and Weihl 4. These include membrane fusion during Golgi reassembly 5, endoplasmic reticulum‐associated degradation 6, NF‐κB activation 7 and the extraction of DNA‐interacting proteins from chromatin 8, 9.

p97 is also associated with inclusion body myopathy with early‐onset Paget disease and frontotemporal dementia (IBMPFD), an autosomal‐dominant multisystem disease 10. A set of mutations in p97 has been shown to cause this disease, which is marked by muscle weakness, early‐onset Paget disease and frontotemporal dementia on the physiological level, and unregulated, excessive enzymatic activity at the molecular level 10. Inhibition of p97 has also been shown to be an effective method of provoking proteotoxic stress in cancer chemotherapy 11.

Each p97 protomer consists of a noncatalytic N‐terminal and two ATPase domains D1 and D2. Both crystallographic and cryo‐electron microscopy studies have shown that in the physiological hexamer, the ATPase domains form planar rings with sixfold symmetry with the N domain either coplanar to the D1 domain or sitting ‘on top’ of the protein 12, 13. The most pronounced movement within p97 has been reported for the N domains. Depending on whether ADP or ATP is bound to the protein, these domains have been observed in a D1 coplanar state (ADP bound) or in an ‘up’ position (ATP bound) 12. The N domain makes interactions with p97 cofactors, which allow p97 to mediate diverse physiological roles 14. These proteins include Fas‐associated factor Faf1 and the Ufd1/Npl4 heterodimer, which play a role in ERAD, and the protein p47, essential to membrane fusion song 5, 6, 15, 16. A large proportion of p97 cofactors, including the three mentioned above, bind ubiquitin and link the ATPase p97 with the ubiquitin system, reviewed by Hanzelmann & Schindelin 17; Stach & Freemont 1.

Both mammalian p97 and its *Saccharomyces cerevisiae* homologue cdc48 unfold proteins in a process that requires ATP hydrolysis, the presence of the Ufd1/Npl4 cofactor and a K48‐linked polyubiquitinated substrate 18, 19. The substrate is unfolded by threading it through the central pore formed by the hexamer 19. Whether the six protomers act in a processive or concerted manner, the nature of the catalytic cycle of this enzyme is less clear than the fate of its substrate. High‐resolution cryo‐EM structures in both ADP‐ and ATP‐bound states were shown to be symmetric, supporting a concerted mechanism 12. On the other hand, it has been shown that the D2 ring can only bind 3 or 4 molecules of ATPγS, suggesting an asymmetry in the p97 hexamer and a processive enzyme mechanism 20. In addition, analysis of the p97 structures submitted to the PDB has identified asymmetry in a number of depositions, significantly in those that contain the D1‐D2 linker region that has been proposed to induce asymmetry 21.

The mechanism by which p97 converts the energy of ATP hydrolysis into mechanical force to unfold proteins has also not yet been determined, but there is evidence of a split‐washer‐type mechanism for its archaeal homologue VCP‐like ATPase (VAT) 22. In its ATP‐bound state, the ATPase domains form planar rings, but in the ADP‐bound state, the 12 ATPase domains form a continuous, helical, split‐washer conformation 22. It has been proposed that the conformational changes from planar to helical conformation provide the mechanical energy for unfolding. Interestingly, there is considerable conservation between p97 and VAT, in terms of both sequence and function. Pairwise structure comparison reveals 53% sequence identity over 254 aligned residues of p97 D2 domain compared to VAT (*z*‐score 25.9 and rmsd of 2.3) (DALI server, 23. The archaeal protein unfolds substrates tagged with the C‐terminal degradation tag ssrA 24. While wild‐type p97 is not active against such substrates, a p97 construct with two‐point mutants in the D1 pore and an N domain deletion effectively unfolds ssrA substrates, suggesting these two ATPases are related mechanistically 25. Furthermore, VAT has been shown to unfold substrates by threading them through the central pore, as also reported for cdc48 19, 26. Similarly, the disaggregase Hsp104, found in lower eukaryotes, threads its substrates through the central pore in a ratchet‐like mechanism 27.

The majority of drug discovery efforts developing p97 inhibitors focus on the D2 domain as it mediates the main catalytic activity. The ATP‐competitive inhibitor CB‐5083 is specific for the D2 active sites, while the well‐characterised allosteric inhibitor NMS‐873 interacts with a region spanning the D1 and D2 domains of adjacent protomers 28, 29. Structural information about the D2 domain, however, which may aid such efforts, is limited to low‐resolution crystal structures in a nonphysiological heptamer, with mutations and deletions in D2 or low‐throughput cryo‐EM 12, 30, 31.

Here, we report the crystal structure of the minimal p97 D2 domain crystallised in a P6_1_ space group closely resembling the VAT helical conformation, suggesting the split‐washer‐type mechanism may be conserved from archaea to metazoans. This crystal system also has potential as a tool for developing novel inhibitors against the p97 D2 ATPase domain, and we describe a fragment‐based screen to identify potential novel p97 chemical ligands. The recent termination of all CB‐5083 clinical trials due to off‐target effects 32 highlights the need to develop novel inhibitors against this promising drug target (ClinicalTrials.gov Identifiers NCT02243917 and NCT02223598).

## Materials and methods

### Molecular biology, protein expression and purification

Fragments of the human p97 gene, coding for the FL protein (amino acids 1‐806), a C‐terminal truncation (1‐764), ND1 (1‐481), D2 domain (463‐764) and the D2 with a C‐terminal extension (463‐806), were amplified by PCR and cloned into the pET47b vector (Novagen/Merck, Darmstadt, Germany). The resultant constructs with N‐terminal polyhistidine tags were expressed in *E. coli* BL21 DE3 gold cells (Agilent, Santa Clara, CA, USA) grown in LB. Cultures were induced at OD_600_ of 0.6 at 20 degrees and grown overnight. Cell pellets were suspended in 500 mm NaCl, 50 mm Tris pH8.0, 0.5 mm TCEP and 10 mm imidazole. Clarified cell lysate was purified by Ni‐affinity followed by size‐exclusion chromatography using a KW804 gel filtration column (Shodex, Tokyo, Japan). Gel filtration buffer was 150 mm NaCl, 20 mm Tris pH7.5 and 0.5 mm TCEP. For protein used in crystallisation, the polyhistidine tag was removed prior to gel filtration using rhinovirus 3C protease. The protease was used at a molar ratio of 1/500 at 4 degrees overnight and not specifically removed as it was used at such a low ratio.

### Protein crystallisation

Crystallisation drops were set up at room temperature in MRC 2‐well plates (Swissci, Neuheim, Switzerland) using a Mosquito robot (TTP Labtech, Melbourn, UK) using 100 nL of 8 mg·mL^−1^ p97 D2 in gel filtration buffer and 100 nL of well solution. Initial crystals grew in: 0.12 m 1,6‐hexanediol; 0.12 m 1‐butanol; 01.2 m 1,2‐propanediol; 01.2 m 2‐propanol; 01.2 m 1,4‐butanediol; 01.2 m 1,3‐propanediol; O.1 m sodium HEPES; 0.1 m MOPS; 12.5% v/v MPD; 12.5% poly(ethylene glycol) 1000; and 12.5% w/v poly(ethylene glycol) 3350 at pH 7.5 (Molecular Dimensions Morpheus D4). Crystals were used to produce a microseed solution using a Seed Bead (Hampton Research) according to the manufacturer's instructions. Optimised crystals were grown in EasyXtal (Qiagen, Hilden, Germany) 15‐well plates (Qiagen) from drops containing 1 μL protein solution, 500 nL microseed solution and 1 μL of 0.08 m l‐Na‐glutamate; 0.08 m alanine (racemic); 0.08 m glycine; 0.08 m lysine HCl (racemic); 0.08 m serine (racemic), 0.08 m Tris, 0.08 m Bicine, 10% v/v MPD; 10% poly(ethylene glycol) 1000; 10% w/v poly(ethylene glycol) 3350 at pH 8.5 (Molecular Dimensions Morpheus H12 diluted with water to 80%). Crystal soaking as part of the XChem experiment was performed according to Collins *et al*. 33. Crystals were soaked for 1 h in 50 mm screening compound at a final DMSO concentration of 10% in the crystal drop before flash‐freezing in liquid nitrogen.

### Crystallography

Crystals were flash‐frozen in liquid nitrogen, and diffraction data were collected at Diamond Light Source beamlines IO3 and I04‐1. Diffraction images were indexed, scaled and integrated using XDS 34. The data set was phased by molecular replacement using part of the structure of 5FTJ and the program phaser 35. The structure was refined using phenix.refine and manual model building using coot 35, 36. Structural information was visualised using PyMOL (The PyMOL Molecular Graphics System, version 1.8; Schrödinger, LLC, New York, NY, USA) and UCSF chimera 37.

### ATPase assay

The ATPase assay was performed using fluorescently labelled ParM on a Clariostar plate reader (BMG) measuring at 540 nm excitation and 585 nm emission. The ParM was prepared and applied in the ATPase assay as described in Kunzelmann and Webb 38. Enzyme concentration was 100 nm with substrate concentrations as indicated. Measurements were made every 2 min, and the increase in fluorescence fitted to a linear regression. Michaelis–Menten kinetics were calculated using prism 7 (GraphPad, San Diego, CA, USA). For the experiments testing sensitivity of p97 constructs to inhibitors CB‐5083 and NMS‐873, 250 μm ATP and 100 μm inhibitor were used.

### Multiangle laser light scattering

Samples of purified protein were applied at a flow rate of 0.5 mL·min^−1^ to a KW804 column (Shodex) pre‐equilibrated in 150 mm NaCl, 20 mm Tris/HCl pH 8.0 and 0.5 mm TCEP and mounted on an Infinity Isocratic Pump (Agilent). A DAWN‐TREOS multiangle laser light scattering detector (Wyatt Technology Corp., Santa Barbara, CA, USA) recorded the scattered light intensity of the column eluent at 3 angles. The protein concentration of the eluent was determined from the change in the refractive index (dn/dc = 0.186) detected by an Optilab rEX differential refractometer equipped with a Peltier temperature‐regulated flow cell, maintained at 25 °C (Wyatt Technology Corp.). The weight‐averaged molecular weight of material contained in the chromatographic peaks was determined with ASTRA (Wyatt Technology Corp.).

### Isothermal titration calorimetry

Isothermal titration calorimetry experiments were performed using an ITC‐200 microcalorimeter (MicroCal/Malvern Panalytical, Malvern, UK) and analysed using ORIGIN software as per the manufacturer's instructions assuming a single‐site binding model. Proteins were dialysed into 150 mm NaCl, 20 mm Tris/HCl pH 8.0, 0.5 mm TCEP and 2% DMSO, and their concentrations were determined using UV/visible spectrophotometry. Small molecules were weighed out and dissolved in the same buffer as the protein. All titrations were performed at 20 °C using 19 injections of 2 μL each.

## Results

### Enzyme kinetics of p97 deletion mutants

**Figure 1:**
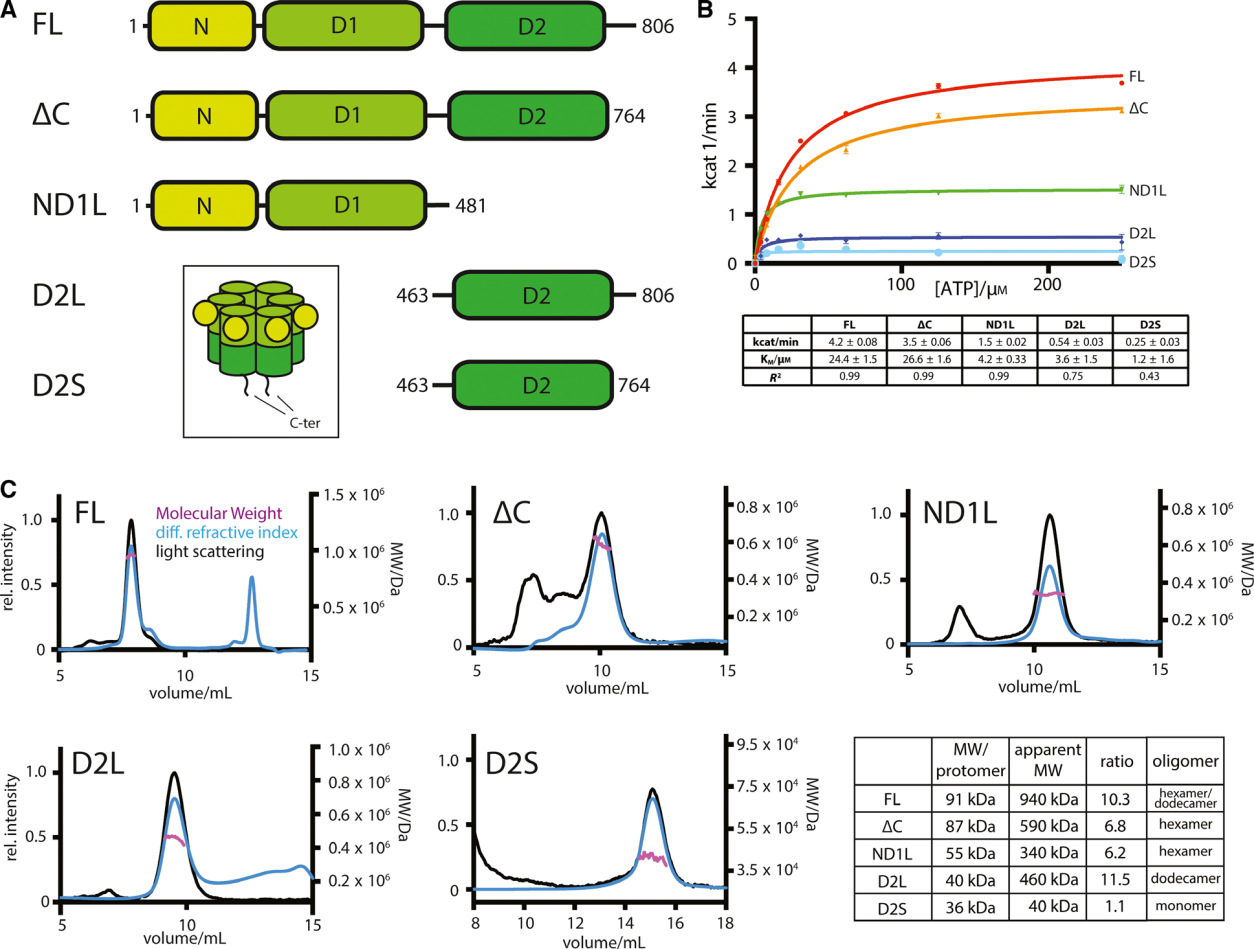
(A) Domain organisation and boundaries of expression constructs used in this study, colour coded according to domain chartreuse: N domain, light green: D1 ATPase domain, dark green: D2 ATPase domain. Inset: schematic of p97 structure. (B) Top: Michaelis–Menten plot of ATPase activity of different p97 constructs used. Below: table with fitted parameters of Michaelis–Menten fits. (C) SEC‐MALLS chromatograms showing light scattering (black), differential refractive index (blue) and fitted molecular weight (purple). The excluded volume of the KW804 column is 6 mL. Below: table comparing calculated protomer molecular weight and observed molecular weight in solution (including standard errors; *n* = 3 technical repeats; Fig. S1) plus most likely oligomeric state of p97 constructs.

In order to obtain a p97 construct containing the D2 domain in its physiological hexameric state, we carried out a p97 deletion analysis. We generated constructs of the full‐length protein (FL), of a C‐terminal deletion (ΔC), of the N domain, the D1 domain and the D1‐D2 linker (ND1L), of the D2 domain with surrounding linker regions (D2L) and of the D2 domain with the D1‐D2 linker (D2S) of p97 that were expressed recombinantly in *Escherichia coli* and purified (Fig. 1A).

The ATPase activity of the proteins was measured, and all five display some degree of activity (Figs 1B and S1). The FL, ΔC and ND1L constructs have activity that fits well with a Michaelis–Menten model and is consistent with previously reported figures 21, 39. The D2L and D2S constructs possess only a fraction of the catalytic activity. Nonetheless, all five proteins, including the newly designed D2 constructs, are catalytically active and thus likely properly folded. While the FL and ΔC have *K*_M_s of approximately 25 μm ATP, the constructs containing only one ATPase domain have considerably lower *K*_M_s, suggesting two intact ATPase rings are a prerequisite for the correct regulation of ATPase activity.

The integrity of the five proteins was also assessed, and their oligomeric states were determined using SEC‐MALLS to identify constructs promising for crystallisation (Fig. 1C). These data suggest an important role for the C‐terminal extension of p97 for oligomerisation of the enzyme. The FL protein has an apparent molecular weight of 940 kDa, more than 10 times larger than the 91 kDa protomer. Given that p97 is known to form highly stable hexameric rings 13, this suggests p97 FL exists in a hexamer/dodecamer equilibrium in solution. It is also unlikely that the hexamers and dodecamers can be resolved separately on the chosen size‐exclusion column, hence why only one peak is visible despite the two oligomeric species present. The ΔC construct elutes later, and its light scattering indicates a molecular weight of 590 kDa, corresponding to a hexamer. Similarly, ND1L constructs also form hexamers. The effect of the C‐terminal extension is particularly clear for the two D2 constructs. The longer construct, which includes the extensions, has an apparent molecular weight of 460 kDa, corresponding to a dodecamer, while the shorter construct is monomeric.

**Figure 2:**
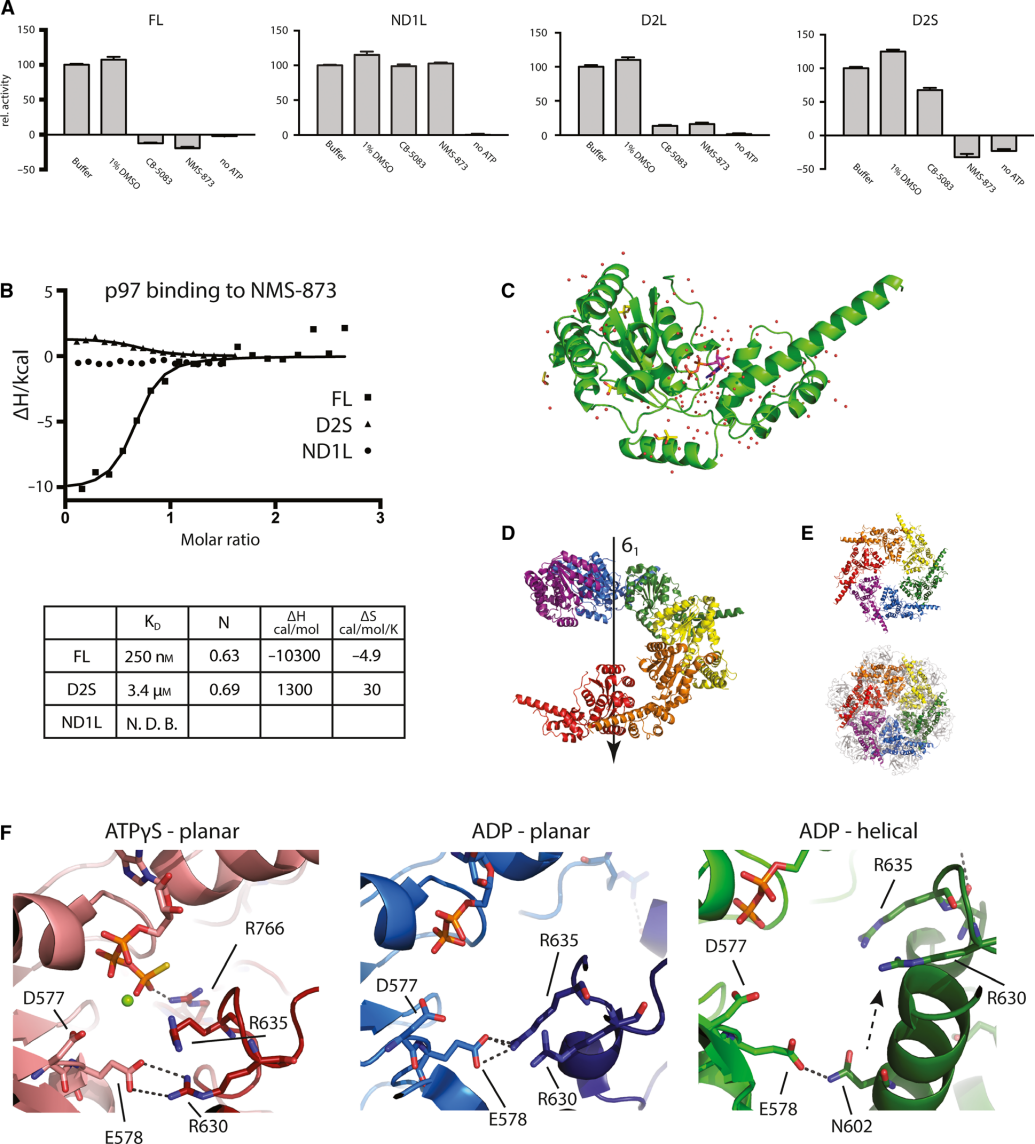
(A) Activity of p97 constructs in the presence of inhibitors, normalised to 100% for buffer only. (B) Binding of NMS‐873 to p97 constructs measured by ITC. Table of binding parameters below. (C) Cartoon representation of one p97 D2S domain from crystal structure with water molecules shown as red dots and ligands shown as sticks. ATP in purple and DMSO and MPD in yellow. (D) Cartoon representation of six consecutive D2S domains along the screw axis. (E) Top: Same 6 D2S domains as in D viewed down the screw axis. Bottom: FL p97 (PDB: http://www.rcsb.org/pdb/search/structidSearch.do?structureId=5FTK) viewed from ‘below’ with the D2 domains closest to the observer. (F) Comparison of interprotomer contacts near the ATP binding site. Cartoon representation of planar p97 in the presence of ATPγS (red, PDB: http://www.rcsb.org/pdb/search/structidSearch.do?structureId=5FTL), ADP (blue, PDB: http://www.rcsb.org/pdb/search/structidSearch.do?structureId=5FTK) and the helical p97 (green). Cartoon representation with key residues and ligands shown as sticks. Magnesium ion shown as green ball.

The sensitivity of the different constructs to two known p97 inhibitors was also measured to ascertain whether these constructs could be used to determine p97 inhibitor complexes (Fig. 2A). As previously reported, the FL protein is efficiently inhibited both by the ATP‐competitive inhibitor CB‐5083 and the allosteric inhibitor NMS‐873. The ND1L construct conversely is insensitive to either inhibitor. As expected, the D2L and D2S are sensitive to both inhibitors.

The binding of the different p97 constructs to the allosteric inhibitor NMS‐873 was measured by isothermal titration calorimetry (ITC). The FL protein binds NMS‐873 with a *K*_D_ of 250 nm, while the affinity of the D2S construct is approximately 10‐fold weaker (Fig. 2B). As expected for an inhibitor specific for the p97 D2 domain, NMS‐873 does not bind the ND1L construct. Given that D2S shows ATPase activity, remains monomeric in solution, and displays some sensitivity to both a D2‐specific allosteric inhibitor and ATP‐competitive one, crystallisation efforts were focused on this construct.

### Crystal structure of p97 D2S shows a helical arrangement

To this end, single well‐diffracting crystals of D2S protein were obtained, and diffraction data were collected and processed up to 1.9 Å (Table S1). The crystal was in the P6_1_ space group, with one ATPase domain bound to ADP per asymmetric unit (Fig. 2C). In addition, each protein molecule was also bound to a single molecule of DMSO and four molecules of MPD from the crystallisation solution. The D2 pore loop could not be resolved and is most likely disordered. The arrangement of molecules along the 6_1_ screw axis (Fig. 2D) somewhat resembles the physiological p97 hexamer. Looking down the screw axis, the arrangement of the symmetry mates is highly similar to the arrangement of the D2 domains in the physiological hexamer when viewed from ‘below’ (Fig. 2E).

This similarity opens up the question as to whether this helical arrangement is purely a crystallisation artefact or of physiological significance. The interprotomer contacts of p97 in this helical conformation were compared to the structures of p97 in a planar conformation, in complex with both ADP (PDB: http://www.rcsb.org/pdb/search/structidSearch.do?structureId=5FTK) and ATP**γ**S (PDB: http://www.rcsb.org/pdb/search/structidSearch.do?structureId=5FTN). Residue E587, part of the glutamate switch of p97, an important regulatory element present in AAA+ proteins, forms a salt bridge with R630 (ATPγS form) or R635 (ADP planar form) in the planar form (Fig. 2F)^40^. The arginine residues are part of the same loop. However, in the helical form, one protomer is moved upwards and these two arginine residues make no interprotomer contacts. Instead, N602 forms an interaction with E587 of the neighbouring protomer. While some interactions are broken in the switch to the helical conformation, new ones are formed.

### Fragment‐based screening using crystals of D2S

The crystal system presented here was used for crystal‐based fragment screening. As the active site of D2S in the crystal is occupied by ADP, and removal of ADP from D2S leads to protein precipitation (data not shown), the aim of the drug screening was to identify compounds that bind D2S away from the active site. These may be developed into allosteric inhibitors, possibly specific for the split‐washer conformation rather than the common and prevalent ATPase active site. Including 40 control DMSO soaks, a total of 527 crystals were used in a crystallographic fragment soaking experiment conducted at the Diamond Light Source XChem facility, and data were collected from 487 crystals. Due to batch to batch variation between protein preparations and difficulties in growing well‐diffracting crystals in plates compatible with the highly automated XChem set‐up, only 58 crystals diffracted to better than 3.0 A. In addition, crystal‐to‐crystal variability in cell dimensions and in the conformation of some surface loops made analysis of the crystals using a pan‐crystal approach (PANDDA) challenging 41.

The data sets were analysed using pandda software, and two hits were found in the same p97 pocket. Strikingly, the crystals with fragments bound diffracted to significantly higher resolution (2.08 and 2.15 Å, respectively) and were the best diffracting crystals of the ones collected. Thus, a manual inspection of all data sets with resolution better than 2.8 Å was carried out and three additional fragments were identified using this approach. It is possible that crystal‐to‐crystal variability in unit‐cell parameters was the most likely cause why PANDDA could not be used to identify these hits.

**Figure 3:**
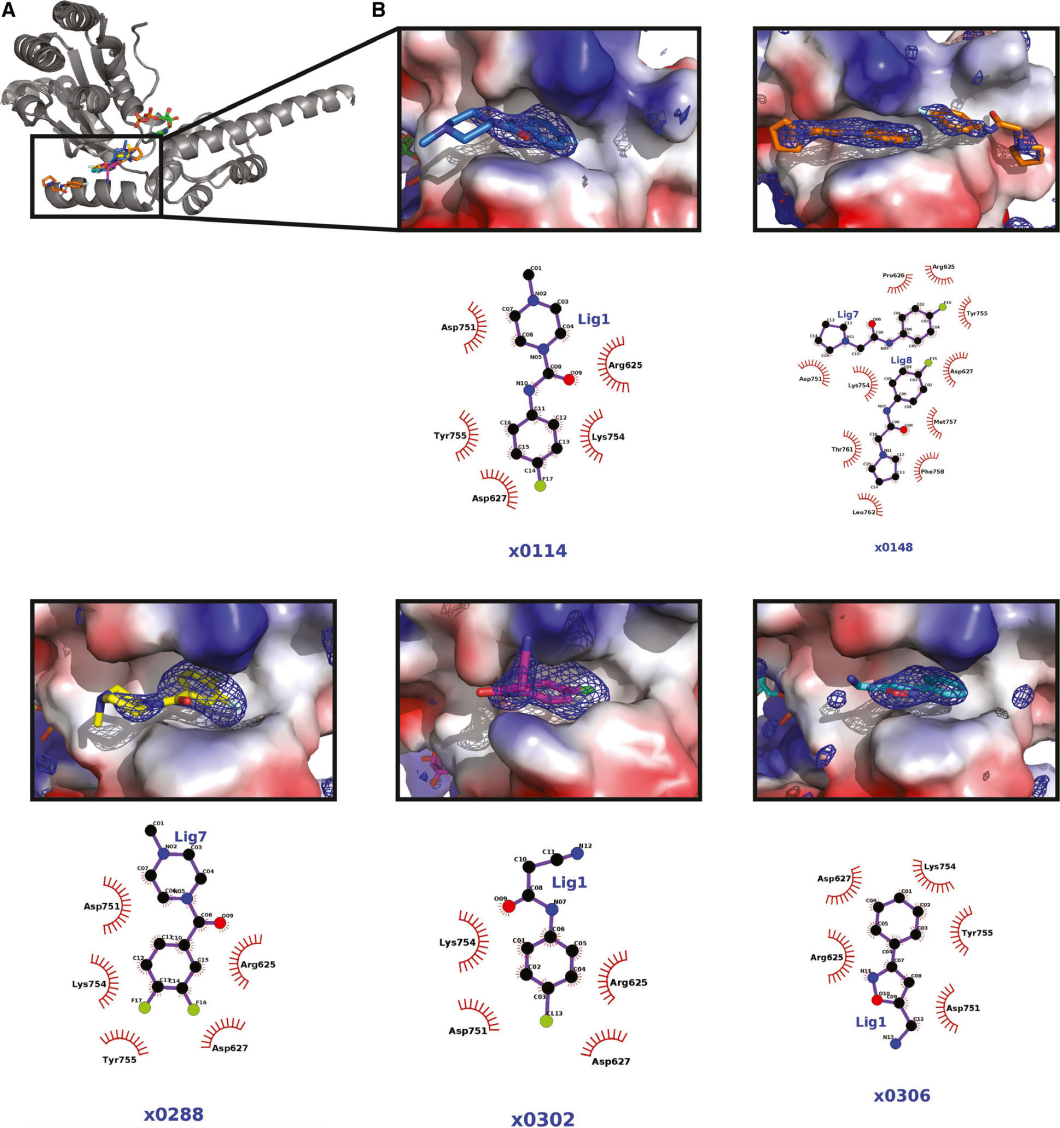
(A) p97 D2S crystal structures of five XChem hits aligned and shown as grey cartoon. ADP shown as sticks in green and XChem hits shown as sticks in same colours as in B. (B) Close‐up of interactions of XChem ligands with D2S domain. D2S shown as surface colour according to charge, ligand shown as sticks and polder map shown as mesh in blue contoured at 3σ. Chemical structure of ligand shown below with interacting protein residues indicated.

All five fragments identified bind in the same groove on the p97 D2 domain (Figs 3 and S2). The fragments have in common phenyl group that fits into the cavity on the protein surface. In four out of the five hits, these phenyl groups are halogenated, and where organofluorine compounds were identified (114, 148, 302 and 306), the fluorine atoms make extensive contacts (moderate‐to‐weak electrostatic interactions) with main chain and side chain atoms of surrounding amino acids Asp627, Tyr 755 and Lys 754. Away from the fragment phenyl ring, electrostatic interactions can be seen with neighbouring Arg625, Asp751 and Lys754 side chains. In the case of the structure for p97 bound to compound ×0148, two copies of the ×0148 fragment are present. One copy is bound in a similar conformation in the same site to the other hits identified. The second copy is bound further downstream of the p97 D2 groove (~ 4.5 Å apart) and interacts with another subset of p97 amino acids. Thr761, Met757 and Phe758 make contacts with the compound's oxygen atom, and the nitrogen atom of the compound's pyrrolidine ring structure hydrogen bonds with the main chain oxygen of Phe758 and the side chain oxygen of Thr761. These observations provide insights on how these potential lead compounds could be extended into the D2 groove.

### Comparison of p97 D2S structure with VAT

The helical parameters of the 6_1_ screw axis are highly similar to a cryo‐EM structure of VAT in a helical oligomerisation state 26. It has been observed that around half of VAT molecules are in a split‐washer conformation when incubated with ADP. To probe whether the helical arrangement found in our p97 D2S structure is related to the VAT split washer, a model was built where the structures of six FL, ADP‐bound, p97 protomers (PDB: http://www.rcsb.org/pdb/search/structidSearch.do?structureId=5FTK) were superimposed on six consecutive D2S symmetry mates along the screw axis (Fig. 3A). Strikingly, this theoretical model of p97, based on the D2S crystal symmetry presented here, overlays well with the published cryo‐EM map of VAT (rmsd = 36.169) 22. While no such split‐washer particles have been observed in cryo‐EM studies of mammalian p97, the high degree of similarity between the VAT structure and the D2S screw axis symmetry suggests that p97 can form a ‘split‐washer’‐type conformation during its ATPase cycle. At the centre of this theoretical model, the D1 of one protomer lines up next to the D2 of its neighbour creating a continuous filament of 12 ATPase domains (Fig. 3A).

## Discussion

p97 is implicated in numerous cellular processes and has been the target of a multitude of biochemical and drug discovery studies 4, 42. Nonetheless, a high‐resolution structure of the main catalytic D2 domain, which may aid drug discovery, has been elusive. In addition, the mechanism of force generation required for substrate protein unfolding is not clear. The data presented here provide some steps towards addressing this. The crystal system presented here has been shown to diffract to high resolution, and its solvent channels allow for the soaking of small molecules. The two compounds identified in the XChem screen have a similar chemical group (phenyl moiety) that points into the groove, suggesting that the binding of this class of molecules to D2 is specific. In summary, our XChem experiment has identified a potential hot spot for ligand binding to p97, with five relatively similar fragment hits providing a starting point for the development of more effective p97 allosteric binders that may have p97 inhibitory activity.

**Figure 4:**
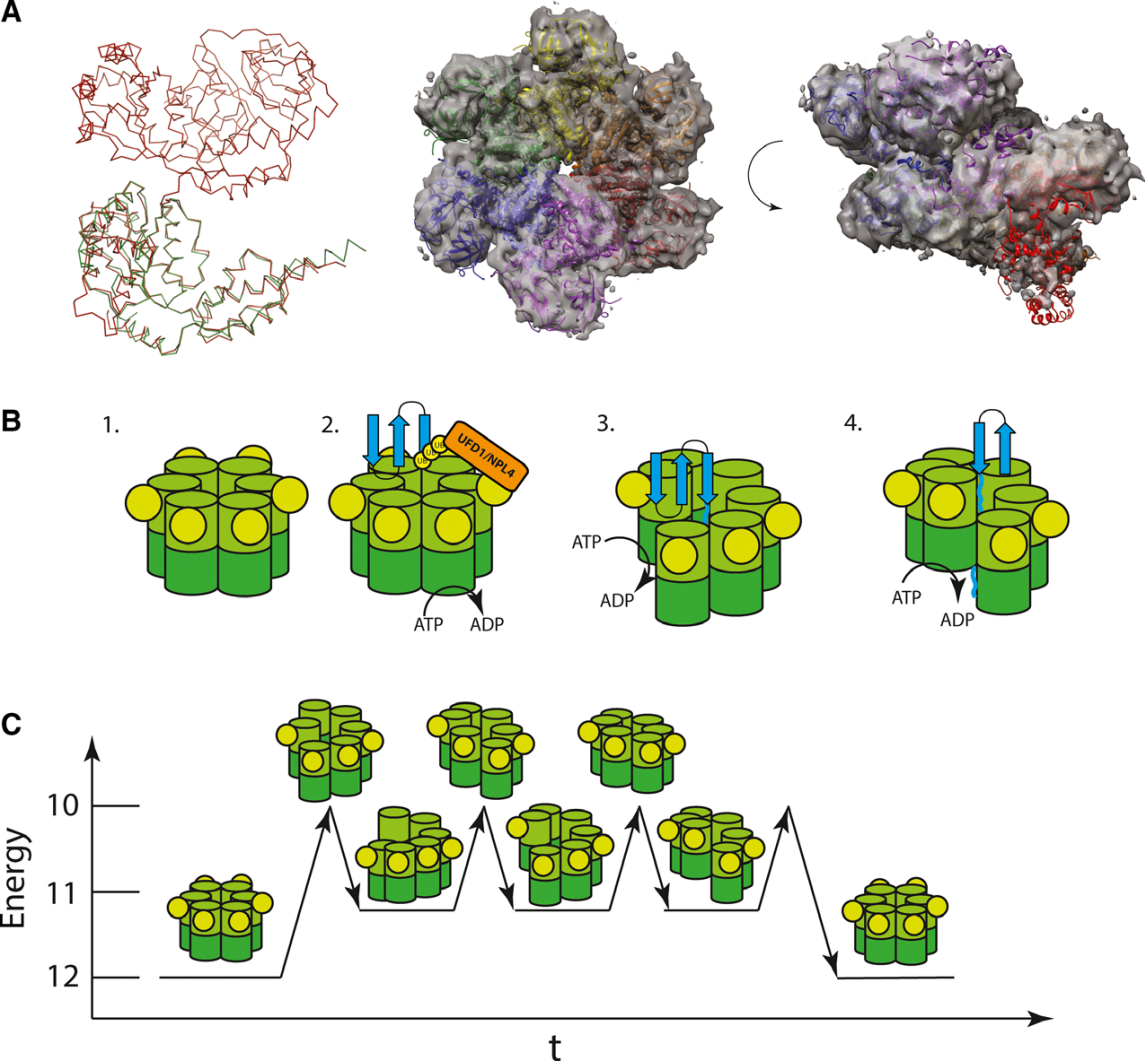
(A) Ribbon representation of one FL p97 protomer (red, PDB: http://www.rcsb.org/pdb/search/structidSearch.do?structureId=5FTK) and D2S crystal structure. Structural model of 6 FL p97 protomers arranged along the P6_1_ screw axis of the crystal as in Fig. 2D, fitted into the electron density of ADP‐bound p97 homologue VAT (EMDB: 3436) shown as transparent surface at contour level 0.0367. (B) Proposed model of p97‐mediated substrate unfolding. A substrate (blue) is threaded through the central pore of p97, being progressively unfolded using the consecutive ATP hydrolysis steps. Each ATP hydrolysis step pushes another protomer upwards causing further unfolding. (C) Energy levels of p97 hexamer throughout unfolding process. The planar conformation being the ground state and the filamentous split washer providing a metastable intermediate. Level of interprotomer contacts indicated on *y*‐axis.

In our new D2S high‐resolution crystal structure, we observe that the helical parameters of the D2S P6_1_ screw axis are highly similar to the split‐washer‐type structure determined by cryo‐EM for VAT 22 (Fig. 4A). Although speculative, it is possible that the high level of similarity between these two structures is of physiological relevance. If so, then our D2S structure allows us to propose a possible model for force generation for p97, which is not dissimilar to the ratchet‐like mechanism reported for Hsp104 27. Given that cryo‐EM studies have never identified p97 in such a conformation, any split‐washer‐type species of p97 is likely short‐lived and therefore not observable by crystallographic or EM analyses.

Based on this speculation, we can propose a mechanistic model for p97. In its resting state, p97 is in a low‐energy planar conformation with twelve inter‐ATPase domain contacts. Upon encountering a ubiquitylated substrate *via* adaptor binding, ATP hydrolysis is stimulated as reported by 18. Hydrolysis in one subunit may cause a conformational change and induce a split‐washer conformation by moving one protomer downwards (Fig. 4B). The movement from a planar conformation to a split washer breaks two inter‐ATPase domain interactions of ~ 1200 Å^2^ in size (Fig. 3E). We propose that this transient conformation is unstable and would likely revert to a more stable planar conformation. However, the twelve‐ATPase domain filament provides a metastable intermediate with eleven intradomain interactions (Fig. 4C). This filament may therefore remain stable long enough for another round of ATP hydrolysis, where the next protomer moves downwards, providing a processive mechanism of action that leads to efficient unfolding or target proteins. Hydrophobic interactions between the D2 pore loop and the substrate protein may further stabilise the helical conformation. However, further structural and biophysical work on p97–substrate complexes would be needed to confirm this speculative p97 mechanistic model.

## Author contributions

LS, XZ and PF devised the study. LS, LM and RM conducted and analysed experiments. AD and FVD provided technology and expertise for the XChem *in crystal* fragment screening. LS, RM and PF wrote and revised manuscript.

## Supporting information


**Fig. S1.** (A) Raw ATPase assay data for FL, deltaC, ND1L, D2L, D2S constructs used in this study, as well as ParM Standards (*n* = 3 technical repeats). (B) Raw activity assay data for FL, ND1L, D2L and D2S constructs.

**Fig. S2** (A) Surface representation in grey of p97 D2S crystal structure with all 5 XChem hits (shown in magenta) superposed in the binding groove. Interacting D2S amino acids are shown in green. (B) Close‐up of superposed XChem hits coloured in magenta and their interacting residues in the p97 D2S domain binding groove (coloured in green: N624, R625, D627, D751,/K754, M757, F758, T761, L762).

**Table S1**. Crystallographic data collection, model, and refinement statistics.
