# Supplementary figures and images for: Crystal structure of the catalytic D2 domain of the AAA+ ATPase p97 reveals a putative helical split‐washer‐type mechanism for substrate unfolding
Source: FEBS Lett. 2019 Nov 22;594(5):933–43. doi: 10.1002/1873-3468.13667 (PMC7154655; doi:10.1002/1873-3468.13667)

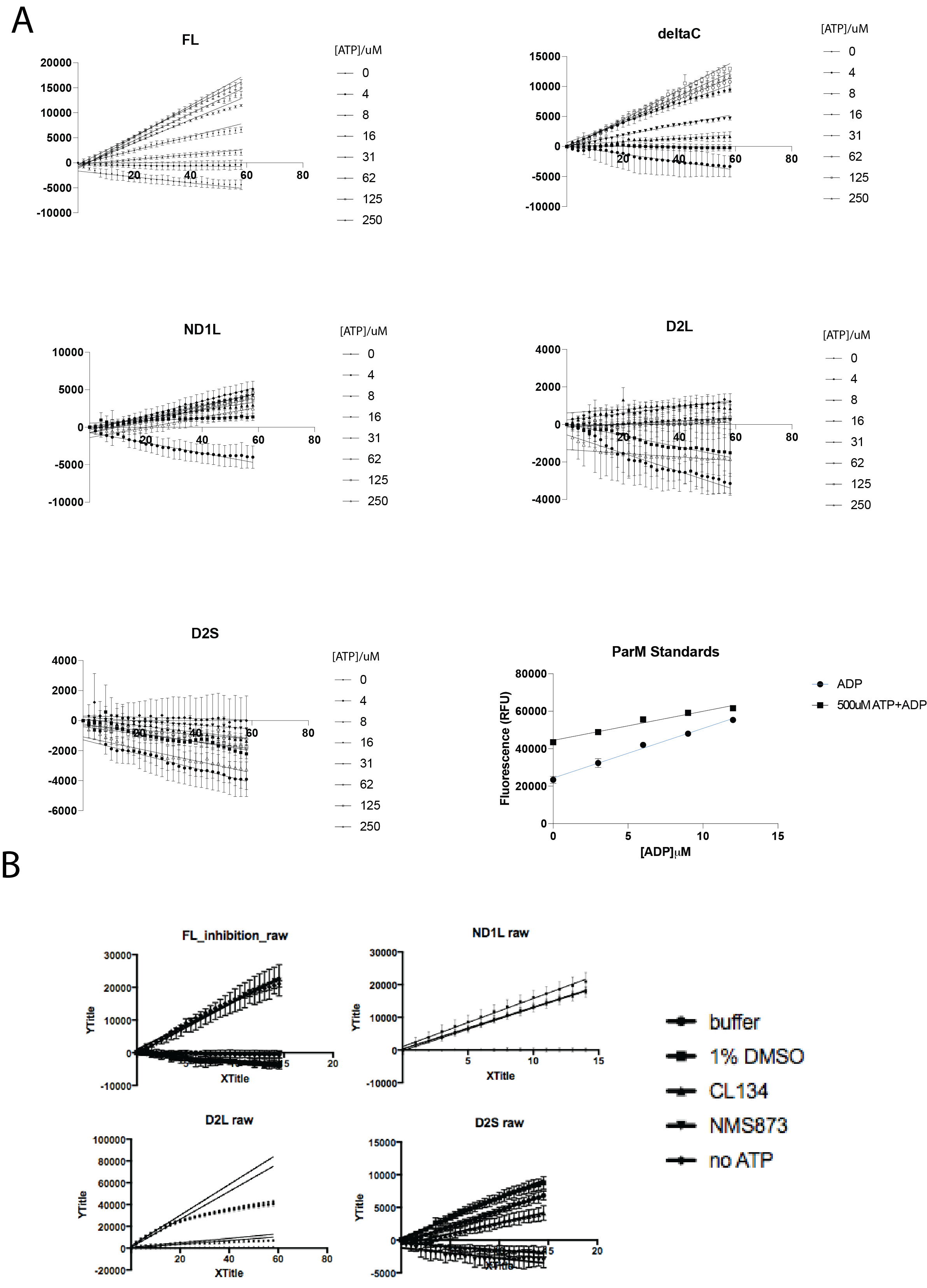

Supplement: Supplementary file 1 — Fig. S1. (A) Raw ATPase assay data for FL, deltaC, ND1L, D2L, D2S constructs used in this study, as well as ParM Standards (n = 3 technical repeats). (B) Raw activity assay data for FL, ND1L, D2L and D2S constructs. [file FEB2-594-933-s001.png]

A

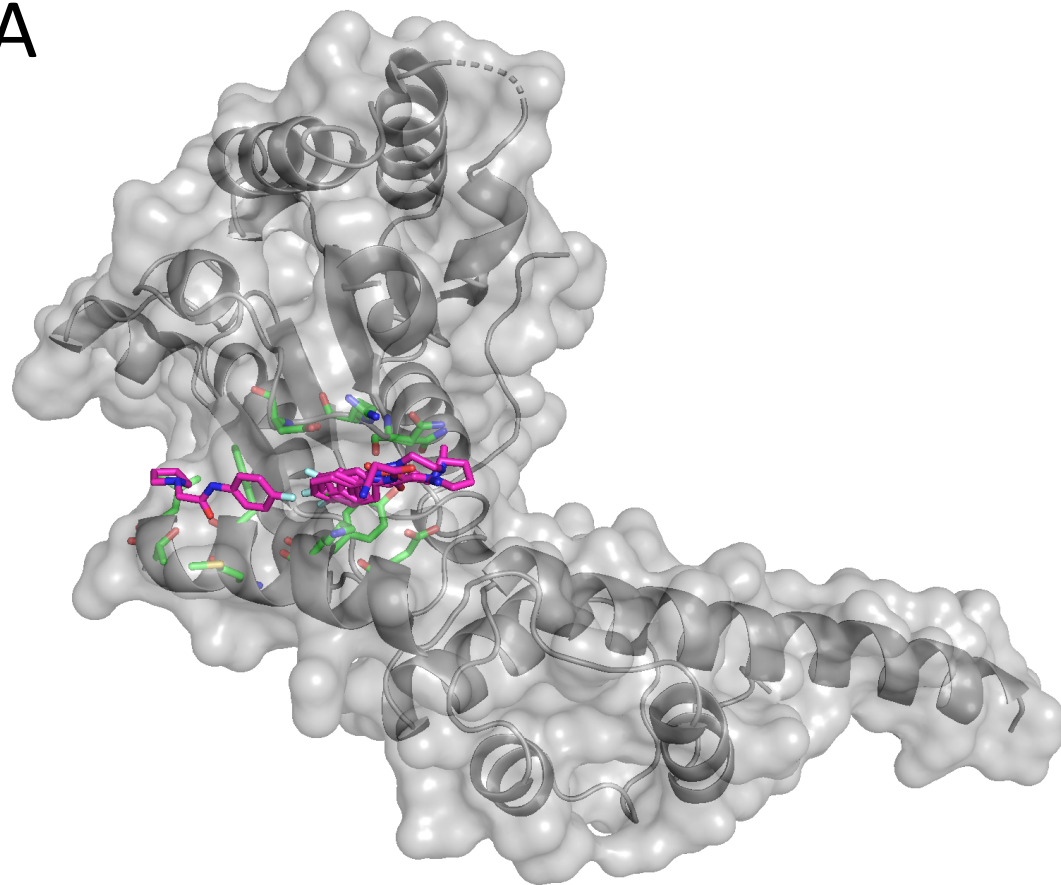

B

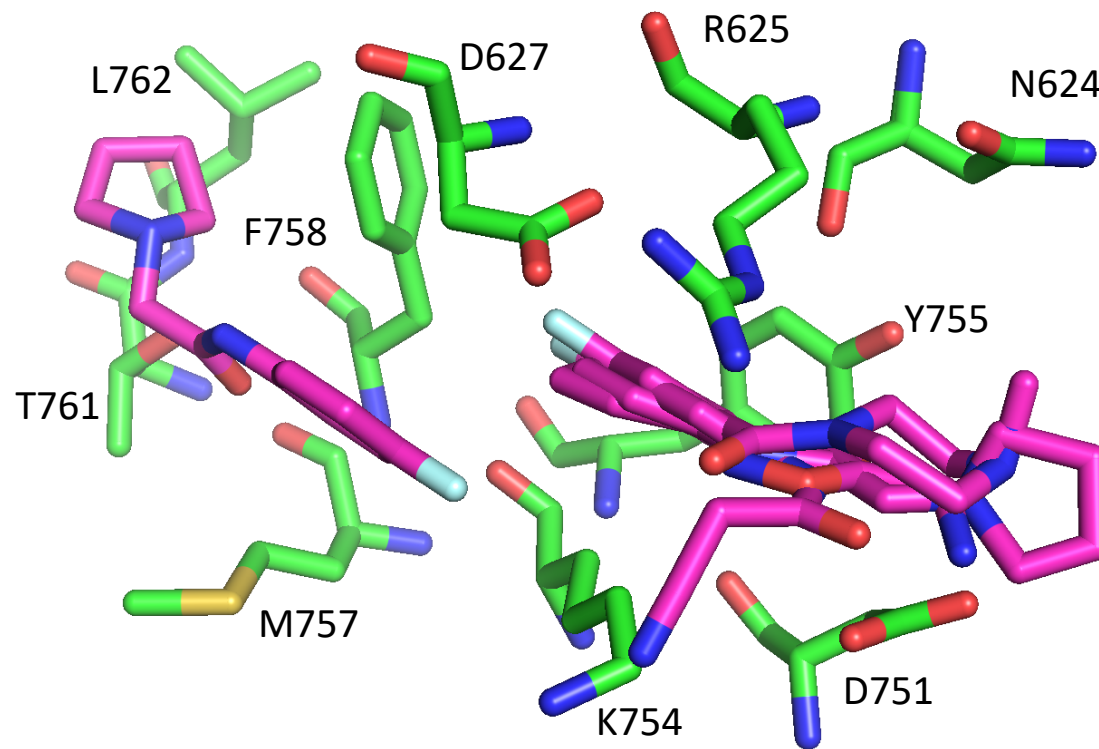

Supplement: Supplementary file 2 — Fig. S2 (A) Surface representation in grey of p97 D2S crystal structure with all 5 XChem hits (shown in magenta) superposed in the binding groove. Interacting D2S amino acids are shown in green. (B) Close‐up of superposed XChem hits coloured in magenta and their interacting residues in the p97 D2S domain binding groove (coloured in green: N624, R625, D627, D751,/K754, M757, F758, T761, L762). [file FEB2-594-933-s002.pdf]
